# K48-Linked Ubiquitination Contributes to Nicotine-Augmented Bone Marrow-Derived Dendritic-Cell-Mediated Adaptive Immunity

**DOI:** 10.3390/vaccines9030278

**Published:** 2021-03-19

**Authors:** Chun Fang Hu, Xiao Yan Liao, Dan Dan Xu, Yi Bin Ruan, Feng Guang Gao

**Affiliations:** 1 Department of Basic Medical Sciences, School of Medicine, Xiamen University, Xiamen 361102, China; 24520141153463@stu.xmu.edu.cn (C.F.H.); 24520181154677@stu.xmu.edu.cn (X.Y.L.); 24520151154264@stu.xmu.edu.cn (D.D.X.); 2 Technology Center, China Tobacco Guizhou Industrial Co., Ltd., Guiyang 550003, China

**Keywords:** adaptive immunity, ubiquitin, nicotine, dendritic cells, bone marrow precursor cells, K48 ubiquitination

## Abstract

K48-linked ubiquitination determining antigen degradation and the endosomal recruitments of p97 and Sec61 plays vital roles in dendritic cell (DC) cross-presentation. Our previous studies revealed that nicotine treatment increases bone marrow-derived dendritic cell (BM-DC) cross-presentation and promotes BM-DC-based cytotoxic T lymphocyte (CTL) priming. But the effect of nicotine on K48-linked ubiquitination and the mechanism of nicotine-increased BM-DC cross-presentation are still uncertain. In this study, we first demonstrated that ex vivo nicotine administration obviously increased K48-linked ubiquitination in BM-DC. Then, we found that K48-linked ubiquitination was essential for nicotine-augmented cross-presentation, BM-DC-based CTL priming, and thereby the superior cytolytic capacity of DC-activated CTL. Importantly, K48-linked ubiquitination was verified to be necessary for nicotine-augmented endosomal recruitments of p97 and Sec61. Importantly, mannose receptor (MR), which is an important antigenic receptor for cross-presentation, was exactly catalyzed with K48-linked ubiquitination by the treatment with nicotine. Thus, these data suggested that K48-linked ubiquitination contributes to the superior adaptive immunity of nicotine-administrated BM-DC. Regulating K48-linked ubiquitination might have therapeutic potential for DC-mediated immune therapy.

## 1. Introduction

Cross-presentation allows dendritic cells (DC) presenting exdogenous antigen and inducing protective immunity against microbe infection and tumors. Bone marrow-derived DC (BM-DC) by granulocyte-macrophage colony-stimulating factor (GM-CSF) and interleukin-4 (IL-4) might result in the absence or the silencing of an immune response in lacking inappropriate encounter with the antigen [1]. Nicotinic acetylcholine receptor (nAChR), which is expressed in BM-DC, epithelial cells, and endothelial cells [2], was found to be involved in splenic nerve activity and enhances plasma cell production [3]. Meanwhile, nicotine was also documented to up-regulate surface molecule expression in DC, augment DC-dependent T-cell priming [4,5,6,7,8]. But, until now, the exact mechanism that nicotine augments DC cross-presentation is still uncertain.

Ubiquitination, a signal for sorting, trafficking, and the removal of membrane proteins via endocytosis [9], regulates innate and adaptive immunity [10,11]. For example, whereas removing K48-linked ubiquitination from mediator of interferon regulatory factor 3 (IRF3) activation (MITA) promotes cellular antiviral responses [10], K48-linked ubiquitination is emerging as a new mechanism for DC cross-presentation [11]. As the proteasomal activity can be efficiently inhibited by the treatment with nicotine [12], a dramatic increase of Akt ubiquitination can be induced in nicotine-treated condition [13]. In our previous studies, despite that ex vivo nicotine treatment was found to increase DC cross-presentation [4,5,6,7,8], the effect of nicotine on K48-linked ubiquitination and the role of K48-linked ubiquitination in nicotine-augmented DC cross-presentation are uncertain.

Cross-presentation, which mainly occurs via endosome-to-cytosol pathway, exports the internalized antigens from the endosomes into the cytosol [14,15]. During this process, p97, an AAA-ATPase located in the endoplasmic reticulum (ER), relocates toward the endosomes and provides the driving force for the transport of misfolded proteins [15,16]. Sec61, another ER component protein, was also documented to relocate from the ER during DC cross-presentation [17,18,19]. Although protein kinase B (Akt)^+^ I-kappa B Kinase α/β (IKKα/β)^+^ Rab5^+^ signalosome is essential for the recruitment of Sec61 toward endosome [19], the ubiquitination of mannose receptor (MR) was also essential for the endosomal relocation of p97 [18]. In our previous studies, the activation of nuclear factor kappa-B (NF-κB) was found to recruit the endosomal relocation of transporter associated with antigen processing (TAP) [20,21]. Hence, despite that the relocation of Sec61 and p97 toward endosomes is essential for DC cross-presentation [22], the exact mechanisms of nicotine-increased endosomal recruitments of Sec61 and p97 need to be clarified.

In this study, we investigated the mechanism by which nicotine augments BM-DC cross-presentation and thereby the superior efficiency of DC-based adaptive immunity by observing the effect of nicotine on BM-DC ubiquitination, the effects of K48-linked ubiquitination on nicotine-increased cross-presentation, the endosomal recruitments of p97 and Sec61, and the superior cytolytic efficiency of DC-activated T cells. Importantly, the treatment with nicotine increases K48-linked ubiquitination of MR in BM-DC was also verified. Our data suggested that K48-linked ubiquitination contributes to the superior adaptive immunity of nicotine-administrated BM-DC. Regulating K48-linked ubiquitination might have therapeutic potential for DC-mediated adaptive immune therapy.

## 2. Materials and Methods

### 2.1. Mice

Pathogen-free C57BL/6 mice (female, 5~6 weeks old) were provided and kept at the Xiamen University Laboratory Animal Center. This study was carried out in strict accordance with the recommendations in the Guide for the Care and Use of Laboratory Animals of the ARRIVE guidelines. Surgeries were performed under ether anesthesia, and efforts were made to minimize suffering.

### 2.2. Reagents and Antibodies

Reagents were purchased from the following companies: Nicotine (N3876) was obtained from Sigma-Aldrich (St. Louis, MO, USA). 4′,6′-diamino-2-fenil-indol (DAPI) was obtained from Vector Laboratories, Inc (Burlingame, CA, USA). Recombinant mouse GM-CSF and IL-4 were obtained from PeproTech (Rocky Hill, NJ, USA). Albumin from chicken egg white (OVA, ovalbumin) was purchased from Beijing Biosynthesis Biotechnology Co., Ltd. (Beijing, China). OVA peptide SIINFEKL of amino acids 257~264 were synthesized by Auspep (Tullamarine, VIC, Australia). MG132 were bought from Selleck Chemicals (Houston, TX, USA). PE-conjugated 25-D1.16, (#141604) was from BioLegend (San Diego, CA, USA). Antibodies to ubiquitin (P4D1, #3936), K48-linkage specific polyubiquitin (D9D5, #8081), K63-linkage specific polyubiquitin (D7A11, #5621), β-actin (13E5, #4970), interferon-γ (IFN-γ) (D3H2, #8455), perforin (E7D8R, #62550), horseradish peroxidase (HRP) conjugate secondary antibody were bought from Cell Signaling Technology (Beverly, MA, USA). Abcam (Cambridge, UK) for MR (15-2, #ab8918), anti-Rabbit IgG (Chromeo 546, #ab60317), anti-Rabbit IgG (Chromeo 488, #ab60314), anti-Mouse IgG (Cy3, #ab97035). CellTrace carboxyfluorescein diacetate succinimidyl ester (CFSE) was obtained from Molecular Probes (Eugene, OR, USA). IFN-γ Elispot Kit was obtained from Dakewei Biosciences (Shenzhen, China). Penicillin-streptomycin-neomycin (PSN) antibiotic mixture was from ThemoFisher Scientific Co., Ltd. (Eugene, OR, USA). RPMI-1640 medium and fetal bovine serum (FBS) was purchased from HyClone (Logan, UT, USA). Cell culture dishes were bought from Thermo Scientific (Shanghai, China). Antibody to p97 (ABIN681178) was obtained from ABNOVA (Taipei, Taiwan). Santa Cruz Biotechnology (Dallas, TX, USA) for siRNAs of ubiquitin (sc-36770), and control siRNA (sc-37007), antibodies to MR (MR5D3), Sec61α (G-20, sc-12322), EEA1 (E-8), Rab5 (D-11, sc-46692), Calnexin (AF-18, sc-23954), and Protein A/G Plus-Agarose, Transfect reagent. Ubiquitin-Proteasome Biotechnologies (Aurora, CO, USA) for bacterial recombinant ubiquitin (#E1610), ubiquitin (K48R-Ub, #E1670), ubiquitin (K63R-Ub, #E1680). The purity of recombinant ubiquitin is about 95% determined by sodium dodecylsulphate polyacrylamide gel electrophoresis (SDS-PAGE). 

### 2.3. Murine Bone Marrow Precursor Cell-Derived Dendritic Cell Preparation

BM-DC was prepared according to previous description [22]. Briefly, the intact femurs and tibias were dissected from 4~6 weeks old C57BL/6 mice. Bone marrow was harvested by repeated flushing with complete RPMI-1640 media (Hyclone). By depleting red blood cells from the bone marrow suspensions, bone marrow mononuclear cells were prepared and incubated in 3.5 cm dishes (Thermo Scientific) at a density of 1 × 10^6^ cells/mL with GM-CSF and IL-4 at the final concentrations of 30 ng/mL and 1 ng/mL, respectively. After 4 d incubation, the media was removed and the cells were further conferred 2 d culture with complete RPMI-1640 media. Then, non-adherent cells were gently washed out with phosphate buffer saline (PBS) and the remaining loosely adherent clusters were referred to BM-DC.

### 2.4. Cell Culture and Cell Line

E.G7-OVA cell line, which was derivative from the C57BL/6 (H-2 Kb) mouse lymphoma cell line EL4 by transfection with the plasmid pAc-neo-OVA, is a model system for studying major histocompatibility complex (MHC)-class-I-restricted responses of cytotoxic T lymphocytes (CTL) in mice. pAc-neo-OVA plasmid carries a complete copy of OVA mRNA and the neomycin (G418) resistance gene. The E.G7-OVA cells were obtained from Shanghai Cancer Institute (Shanghai, China). Cells were cultured in RPMI-1640 media with 10% FBS at 37 °C in 5% CO_2_ in the presence of PSN antibiotic mixture. Cultures can be maintained by addition or replacement of fresh medium.

### 2.5. Ubiquitin siRNA Transfection

siRNA transfection was performed according to the guideline of the manufacturer and our previous description [19]. Briefly, buffer A and B were respectively prepared by diluting 2–8 µL of siRNA duplex (20–80 pmols siRNA) or transfection reagent (sc-29528) into 100 µL siRNA transfection medium (sc-36868). Then, buffer C was completed by gently mixing and incubating buffer A and B for 45 min at room temperature. After washing with transfection medium (sc-36868), the cells were overlaid with siRNA transfection medium containing buffer C and incubated for 7 h. After incubation, normal medium containing 20% FBS was replenished and the cells were conferred 18~24 h incubation. After that, the medium was removed and the cells were further cultured for 3 d. All siRNA sequences are not provided by Santa Cruz, as stated in their datasheets: “siRNA (m) is a pool of 3 target-specific 19–25 nt siRNAs designed to knock down gene expression.” The effect of indicative siRNA in BMPC was validated in supplementary data.

### 2.6. Murine Bone Marrow Precursor Cell-Derived Dendritic Cell Treatment

To determine the effect of nicotine on DC ubiquitination, BM-DC was stimulated with nicotine (10^−7^ mol/L) for 12 to 16 h. To dissect the role of K48-linked ubiquitination in nicotine-increased BM-DC cross-presentation and the endosomal recruitments of p97/Sec61, ubiquitin-deficient and control cells were conferred nicotine (10^−7^ mol/L) stimulation in the presence of K48R-Ub, K63R-Ub, or wild type (WT) ubiquitin (30 × 10^−6^ mol/L) for 12 to 16 h. After washes, the cells were further incubated with ovalbumin (50 μg/mL) for 5~6 h. 

### 2.7. Murine Bone Marrow-Derived Dendritic Cell Adoptive Transfer

To investigate the role of K48-linked ubiquitination in nicotine-increased BM-DC mediated adaptive immunity, ubiquitin deficient and control cells were incubated with K48R-Ub (30 × 10^−6^ mol/L) and stimulated with nicotine (10^−7^ mol/L). After washes, the cells were further incubated with ovalbumin (50 μg/mL) for 5~6 h. Then, the cell was washed with PBS to remove potential antigen. 1 × 10^5^ BM-DC was intraperitoneally transferred into approx. 6-week-old C57BL/6 recipients. 5~7 d after adoptive transfer, splenocytes or lymph nodes from the recipients were prepared and further conferred enzyme-linked immunospot assay (Elispot), Western blot analyses, and cytolytic assay, respectively.

### 2.8. Antigen-Specific Interferon-γ Enzyme-Linked Immunospot Assay

To investigate the role of K48-linked ubiquitination in nicotine-increased BM-DC mediated T-cell priming, Ag-specific IFN-γ Elispot assay was performed [22]. Briefly, 5~7 d after adoptive transfer, 5 × 10^5^ per well lymphocyte from the recipients was prepared and transferred into IFN-γ antibody pre-coated plate. Then, the cells were further re-stimulated with peptide (SIINFEKL) at the final concentration of 4 µg/mL for 16~24 h. The spots were developed and the data were presented as Spot Forming Units per million cells. 

### 2.9. Labeling Cells with CellTrace Carboxyfluorescein Diacetate Succinimidyl Ester

E.G7-OVA cell was labeled with CFSE according to the previous description [23]. Briefly, E.G7-OVA cell was re-suspended in pre-warmed PBS/0.1% bovine serum albumin (BSA) at the final concentration of 1 × 10^6^ cells/mL. Then, CellTrace CFSE was added at the final concentration of 2 µM, and incubated at 37 °C. After that, the dye was removed with centrifugation and the cell was cultured in pre-warmed medium and referred to as target cell in antigen specific CTL cytotoxicity assay.

### 2.10. Cytotoxic T Lymphocyte Cytolytic Assay

CTL cytotoxicity assay was performed according to the published methods [23]. Briefly, the recipients of the adaptive transfer were sacrificed and the spleen was removed. Then, effector cell was prepared by depleting red blood cell from the splenocyte suspension. The effector cells were re-stimulated with SIINFEKL peptide at the final concentration of 4 µg/mL. E.G7-OVA cells (tumor cells with specific expression of OVA) labeled with CFSE was used as target cell. The effector cell was incubated with target cell at the ratio of 2:1 or 5:1, respectively. After 8 h incubation, the fluorescence value of CFSE was analyzed by flow cytometry. An equal number of target cell or effector cell was cultured alone to determine the spontaneous lysis of the cell. The cytolytic capacity of Ag-specific effect cell was calculated according the following formula [23]:

$$\begin{array}{ll} \%Cytotoxicity & =\frac{Target\;Spontaneous\;+\;Effector\;Spontaneous\;-\;Experimental}{Target\;Spontaneous\;+\;Effector\;Spontaneous}\\ & \;\;\times 100\% \end{array}$$

### 2.11. Co-Immunoprecipitation and Immunoprecipitation

Co-immunoprecipitation (Co-IP) and immunoprecipitation (IP) was performed according to previous description [19]. Briefly, BM-DC stimulated with nicotine was harvested in the presence of MG132 (20 × 10^−6^ mol/L) and lysed in the buffer of Pierce™ Co-Immunoprecipitation Kit (Cat. 26149). Then, the lysate was incubated with 20 μL/mL protein A/G agarose beads for 1 h for pre-clear. After that, the supernatant was incubated with ubiquitin or MR primary antibody in *radio*-*immunoprecipitation* *assay* (RIPA) buffer overnight at 4 °C, and further followed by the addition of 20 μL/mL protein A/G agarose beads. After thoroughly washing with RIPA buffer, the immunoprecipitate was centrifuged and re-suspended in SDS sample buffer.

### 2.12. Western Blots

Western blot analysis was performed according to previous description [19]. Briefly, the cellular proteins were extracted and loaded onto 6~8% SDS-PAGE. After 120 min electrophoresis with 80 volt, the proteins were transferred onto PVDF membrane. Blocking was performed by incubation with 5% fat-free milk in TBST. The membrane was incubated with primary antibody at 4 °C overnight with 1:1000 dilutions. After washing six times with TBST (for 10 min each), the membrane was further incubated with corresponding HRP-conjugated secondary antibody at room temperature. The bound secondary antibody was visualized using enhanced chemiluminescence ECL (Advansta, CA, USA). β-actin was used as a loading control. Protein level was quantified by ImageJ software and presented as related integrated density (RID).

### 2.13. Confocal Immunofluorescent Microscope

To investigate the role of K48-linked ubiquitination in nicotine-increased BM-DC cross-presentation and the endosomal recruitments of p97 and Sec61, BM-DC was performed immunofluorescent observation according to previous description [23]. Briefly, BM-DC was fixed in 2% paraformaldehyde (PFA) and permeabilized with 0.2% saponin. Then, the cell was blocked, washed, and stained with primary antibodies over-night at 4 °C. After washes, the cell was incubated with fluorescence-conjugated secondary antibodies for 1 h at 37 °C. DAPI counterstaining was performed to visualize the nuclei. Images were acquired on Olympus FluoView FV1000 confocal microscope with oil immersion objective at the wavelength of 488 nm.

### 2.14. Statistical Analysis

All data were expressed as average of experimental data points, and standard error means were determined using the calculated standard deviation of a data set divided by the number of data points within the data set. Statistical significance was assessed by one-way or two-way ANOVA with the Newman-Keuls post-test, with a value of *p* < 0.05 considered statistically significant. No randomization or exclusion of data points was used. Sample sizes were chosen according to previous experience and preliminary studies to ensure adequate power. 

## 3. Results

### 3.1. The Treatment with Nicotine Increases K48-linked Ubiquitination in Bone Marrow-Derived Dendritic Cells

**Figure 1:**
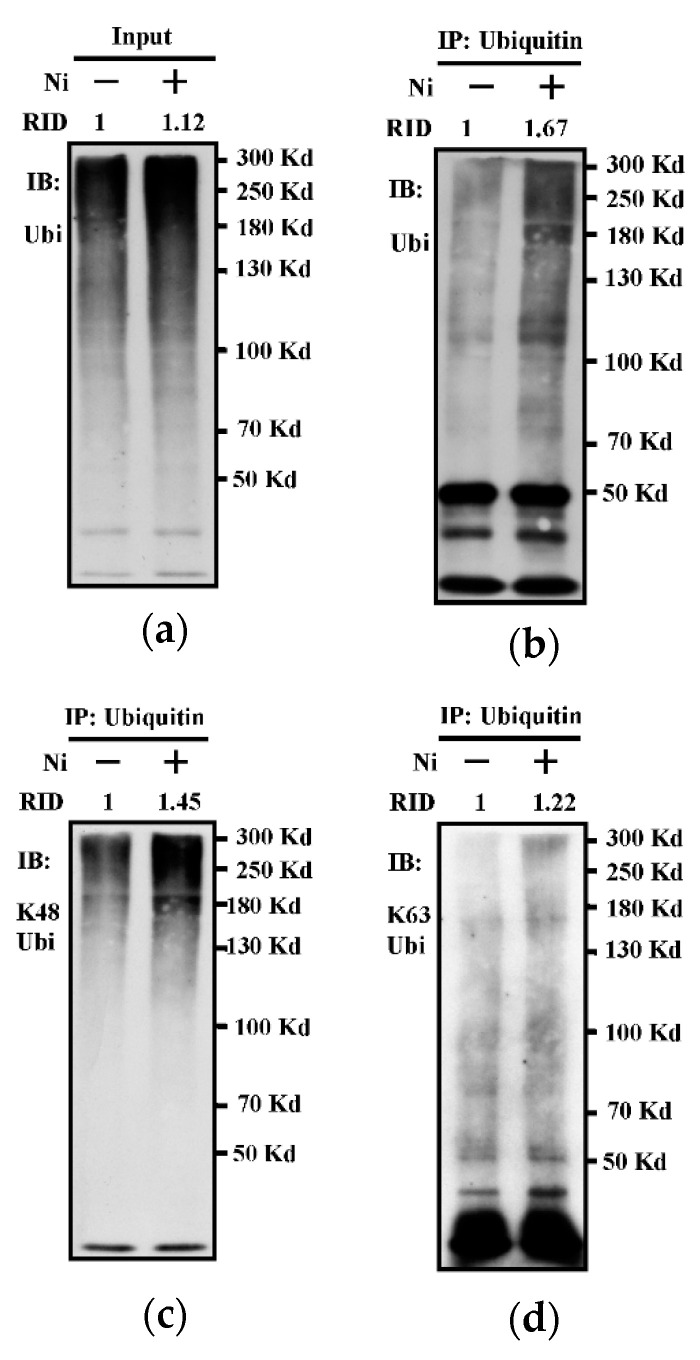
The treatment with nicotine increases K48-linked ubiquitination in bone marrow-derived dendritic cells. (**a**–**d**) Murine BM-DC was incubated with nicotine (10^−7^ mol/L) for 12~16 h in the presence of MG132 (50 × 10^−6^ mol/L). Whole cellular protein was extracted and used as input control (**a**). The levels of ubiquitination (**b**), K48-linked ubiquitination (**c**), and K63-linked ubiquitination (**d**) were determined by IP with indicated antibodies. Protein level was quantified by ImageJ software and presented as related integrated density (RID). One representative from three independent experiments is shown. Ni: nicotine; Ubi: ubiquitination; K48 Ubi: K48-linked ubiquitination; K63 Ubi: K63-linked ubiquitination; IP: immunoprecipitation; IB: immunoblotting.

Cross-presentation occurs in vacuolar and endosome-to-cytosol pathway, degrading antigens within endosomes by lysosomal proteases or in the cytosol by cytosolic proteinase, respectively [10,14,15]. In our previous studies, nicotine was found to increase BM-DC cross-presentation [4,5,6,7,8,24]. Further studies revealed that the inhibition of ubiquitination impairs DC’s cross-presentation [22]. We wonder whether the process of ubiquitination is involved in nicotine-increased DC cross-presentation. To address this issue, we incubated BM-DC with nicotine, and assessed the effect of nicotine on BM-DC ubiquitination by IP. As shown in Figure 1, whereas there was no obvious increase of ubiquitined protein in whole cellular extracts (Figure 1a), an about 67% increase of ubiquitinated protein can be monitored in the output of ubiquitin antibody-anticipated IP (Figure 1b). Similarly, nicotine-increased K48-linked ubiquitinated protein can be easily observed in these output (Figure 1c). Interestingly, when K63-linked ubiquitination was analyzed, no obvious increase can be achieved (Figure 1d). The assessment of K48-linked or K63-linked ubiquitination in whole cellular extracts also revealed the similar results (Supplementary Figure S1). All these data indicate that the treatment with nicotine efficiently increases K48-linked ubiquitination in BM-DC.

### 3.2. K48-linked Ubiquitination Contributes to Nicotine-Increased Cross-Presentation in Bone Marrow-Derived Dendritic Cells

**Figure 2:**
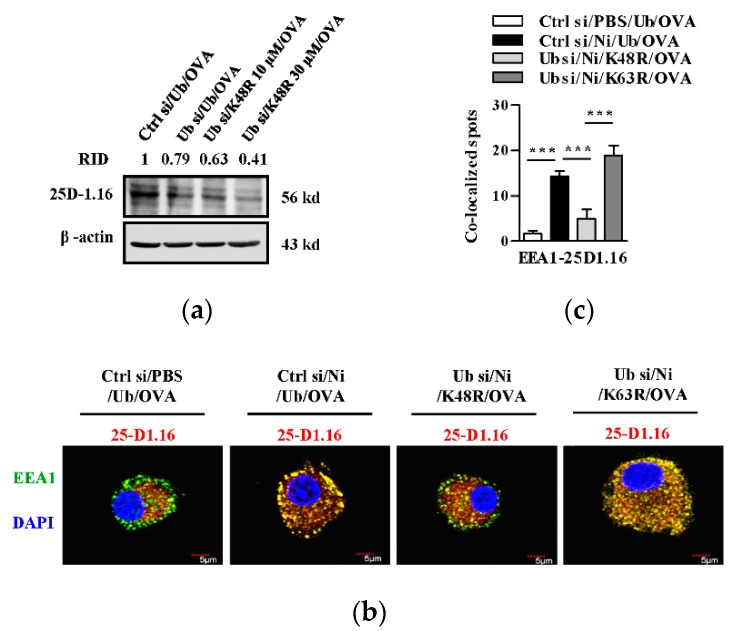
K48-linked ubiquitination contributes to nicotine-increased cross-presentation in bone-marrow-derived dendritic cells. (**a**–**c**) Murine ubiquitin-deficient and control BM-DC were stimulated with PBS (**a**) or nicotine (10^−7^ mol/L) (**b**) for 12~16 h. Then, the cells were replenished with K48R-Ub, K63R-Ub, or WT-Ub (30 × 10^−6^ mol/L or indicated concentration) prior to further incubation with ovalbumin (50 μg/mL). Cross-presented OVA was assessed by Western blot analyses (**a**) or confocal microscope (**b**,**c**). Cross-presented OVA was stained with 25-D1.16 (red) and endosome was stained with EEA1 antibody (green) respectively; nuclei were counterstained with DAPI (blue). The co-localized spots of 25-D1.16 with EEA1 were counted and analyzed (**c**). For Western blot analyses, β-actin was used as an internal control. Protein level was quantified by ImageJ software and presented as related integrated density (RID). Data are presented as the mean ± SEM, *** *p* < 0.001, one-way ANOVA with Newman-Keulspost test. One representative from three independent experiments is shown. Ni: nicotine; Ub: ubiquitin; si: siRNA; EEA1: early endosome marker; 25D1.16: antibody specific to SIINFEKL-H2Kb complex; OVA: ovalbumin.

Lysosome and endosomes are responsible for presenting internalized antigen on MHC II molecules or on MHC I molecules, respectively [17]. We first incubated ubiquitin-deficient and control BM-DC with K48R-Ub and assessed the role of K48-linked ubiquitination in DC cross-presentation. Given that siRNA transfection efficiently decreased ubiquitin expression (Supplementary Figure S2), the deficiency of ubiquitin attenuated antigen loading induced cross-presentation (Figure 2a). As MR is an important receptor for DC cross-presentation [21] and nicotine increases K48-linked ubiquitination (Figure 1), we wonder whether K48-linked ubiquitination plays important roles in DC cross-presentation. We incubated nicotine-treated, ubiquitin-deficient DC with K48R-Ub and found that K48-linked ubiquitination of MR was inhibited by K48R-Ub incubation (Supplementary Figure S3). Interestingly, the replenishment with K48R-Ub also weakened cross-presented OVA (Figure 2a). We then incubated ubiquitin-deficient and control cells with K48R-Ub or K63R-Ub and evaluated the effect of K48-linked ubiquitination in nicotine-increased cross-presentation. As antigen-containing endosome is the compartment of MR-mediated cross-presentation [16,17], we next observed cross-presented OVA by confocal microscope with endosome marker and cross-presented OVA staining. EEA1 is the marker of endosome and 25D1.16 antibody specific to SIINFEKL-H2Kb complex. Whereas nicotine treatment increased the co-localized spots of cross-presented OVA with EEA1, the replenishment with K48R-Ub obviously decreased these co-localized spots (Figure 2b,c). Contrary to K48R-Ub, K63R-Ub had no inhibitory effect on these co-localized spots (Figure 2b,c). When endosome was stained with Rab5 antibody, K48R-Ub-decreased cross-presented OVA can also be derived (Supplementary Figure S4). As nicotine-increased K48-linked ubiquitination of MR was efficiently inhibited by the replenishment with K48R-Ub (Supplementary Figure S3), all these data indicate that K48-linked, but not K63-linked ubiquitination, plays pivotal roles in nicotine-increased cross-presentation in BM-DC.

### 3.3. K48-Linked Ubiquitination Is Essential For Nicotine-Augmented Dendritic Cell-Based CTL Priming

**Figure 3:**
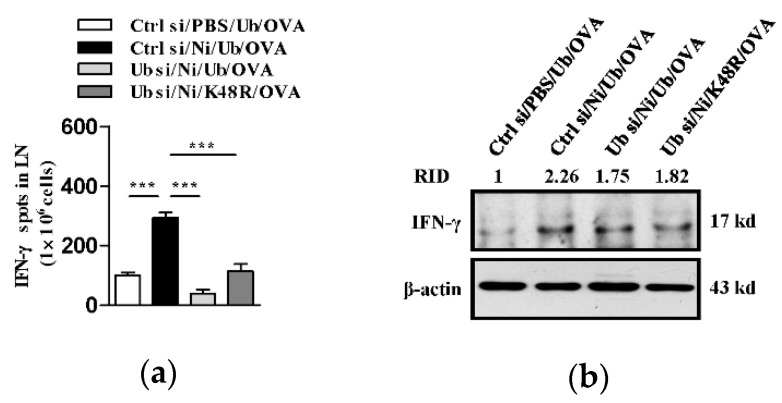
K48-linked ubiquitination contributes to nicotine-augmented dendritic cell-based CTL priming. (**a**,**b**) Murine ubiquitin deficient and control BM-DC were stimulated with nicotine (10^−7^ mol/L) for 12~16 h. Then, the cells were replenished with K48R-Ub or WT-Ub (30 × 10^−6^ mol/L) and further incubated with ovalbumin (50 μg/mL) for 5~6 h. After that, 1 × 10^5^ BM-DC was intraperitoneally transferred into C57BL/6 recipients. 5~7 d after adoptive transfer, splenocytes and lymph nodes of the recipients were prepared. Antigen-specific CTL in the lymph nodes was determined by IFN-γ Elispot assay (**a**). IFN-γ expression in splenocytes was determined by Western blot analyses (**b**). For Western blot analyses, β-actin was used as an internal control. Protein level was quantified by ImageJ software and presented as related integrated density (RID). One representative from three independent experiments is shown. Data were presented as the mean ± SEM of replicates from one experiment. *** *p* < 0.001, one-way ANOVA with Newman-Keulspost test. Ni: nicotine; Ub: ubiquitin; si: siRNA; OVA: ovalbumin.

Antigen presenting cells, such as DC, uptake and process antigens, and present them to T cells to prime series of immune response [14]. Apart from cross-presentation (Figure 2), we assessed the role of K48-linked ubiquitination in nicotine-augmented DC-based CTL priming. Whereas the treatment with nicotine increased about 193.7% antigen-specific IFN-γ spots, the replenishment with K48R-Ub ubiquitin nearly completely abolished nicotine’s effect on CTL priming, with about 79.4% inhibitory rate (Figure 3a). The evaluation of IFN-γ in splenocytes of the recipients demonstrated that K48R-Ub-attenuated nicotine up-regulated IFN-γ expression (Figure 3b). Given that K48R-Ub efficiently inhibited nicotine-augmented K48-linked ubiquitination of MR (Supplementary Figure S5), above data indicate that K48-linked ubiquitination of MR is essential for nicotine-augmented BM-DC-based CTL priming.

### 3.4. K48-Linked Ubiquitination Contributes to Nicotine-Increased Superior Cytolytic Capacity of Dendritic Cell-Activated T Cells

**Figure 4:**
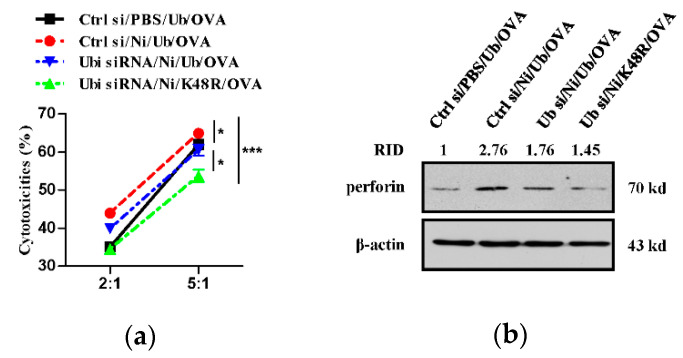
K48 ubiquitination contributes to nicotine-increased cytolytic activities of dendritic cell-activated T cells. (**a**,**b**) Murine ubiquitin deficient and control BM-DC were stimulated with nicotine (10^−7^ mol/L) for 12~16 h. Then, the cells were replenished with K48R-Ub or WT-Ub (30 × 10^−6^ mol/L) and further incubated with ovalbumin (50 μg/mL) for 5~6 h. Then, 1 × 10^5^ BM-DC was intraperitoneally transferred into C57BL/6 recipients. 5~7 d after adoptive transfer, splenocytes of the recipients were prepared and the cytolytic activities were assessed by incubation of effector cells with CFSE-labeled E.G7-OVA target cells. CFSE value was analyzed by flow cytometry, and the cytolytic activities were calculated (**a**). Perforin expression in the splenocytes of the recipients was determined by Western blot analyses (**b**). For Western blot analyses, β-actin was used as an internal control. Protein level was quantified by ImageJ software and presented as related integrated density (RID). One representative from three independent experiments is shown. Data were presented as the mean ± SEM of replicates from one experiment. * *p* < 0.05, *** *p* < 0.001, two-way ANOVA with Newman-Keulspost test. Ni: nicotine; Ub: ubiquitin; si: siRNA; OVA: ovalbumin.

Recently, IL-15-treated and nicotine-stimulated BM-DC are emerging as better alternatives for inducing antigen-specific CTL priming [23]. To assess the effect of K48-linked ubiquitination on nicotine-increased cytolytic capacity of BM-DC activated T cells, we incubated the effector cell of the recipients with CFSE-labeled target E.G7-OVA cells. Whereas ex vivo nicotine treatment efficiently augmented the cytolytic activities of BM-DC-activated splenic T cells, K48R-Ub replenishment abrogated nicotine’s effect on BM-DC activated T cells. The replenishment with WT-Ub partly reversed K48R-Ub decreased the cytolytic activities (Figure 4a). As perforin’s pore-forming activity is necessary for CTL delivery of proapoptotic serine proteases, granzymes, into the cytosol of infected or cancerous target cells [25], we next evaluated perforin expression. As shown in Figure 4b, whereas the treatment with nicotine increased perforin expression (Figure 4b), K48R-Ub abrogated nicotine’s effect on perforin up-regulation. Given that K48R-Ub replenishment efficiently inhibited nicotine-augmented K48-linked ubiquitination of MR (Supplementary Figure S3), these data indicate that K48-linked ubiquitination play vital roles in nicotine-augmented cytolytic activities of BM-DC-activated T cells.

### 3.5. K48-Linked Ubiquitination Facilitetes Nicotine-Increased Endosomal Relocation of p97 in Dendritic Cells

**Figure 5:**
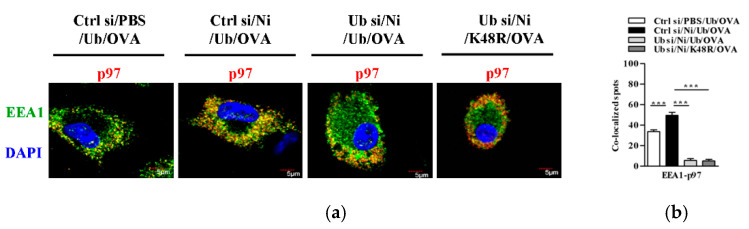

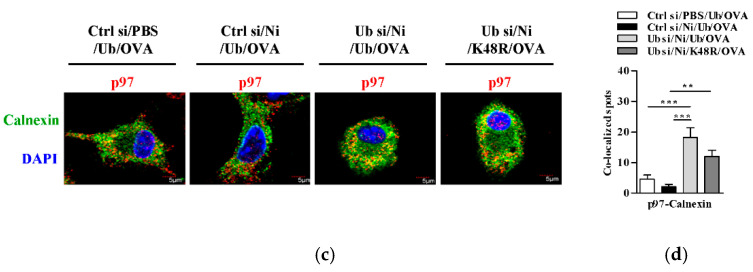
K48-linked ubiquitination is essential for nicotine-augmented endosomal relocation of p97 in bone marrow-derived dendritic cells. (**a**,**b**) Murine ubiquitin-deficient and control BM-DC were stimulated with nicotine (10^−7^ mol/L) for 12~16 h. Then, the cells were replenished with K48R-Ub or WT-Ub (30 × 10^−6^ mol/L) and further incubated with ovalbumin (50 μg/mL) for 5~6 h. The endosomal relocation of p97 (**a**) from the endoplasmic reticulum (**b**) was determined by confocal microscope with EEA1, calnexin, and p97 antibody staining. EEA1 (**a**), calnexin (**b**) were stained green; p97 was stained red; nuclei were counterstained with DAPI (blue). The co-localized spots of p97 with EEA1 (**c**) and p97 with calnexin (**d**) were counted and analyzed. Data are presented as the mean ± SEM, ** *p* < 0.01, *** *p* < 0.001, one-way ANOVA with Newman-Keulspost test. One representative from three independent experiments is shown. EEA1: early endosome marker; Calnexin: endoplasmic reticulum marker; Ni: nicotine; Ub: ubiquitin; si: siRNA; OVA: ovalbumin.

In the process of cross-presentation, a group of proteins, such as p97, translocate to endosomes to facilitate the transport of internalized antigen from endosomes to cytosol [15,16,22]. To verify the role of K48-linked ubiquitination in nicotine-increased DC cross-presentation, we incubated BM-DC with K48R-Ub and assessed the translocation of p97 from the ER toward endosomes. As shown in Figure 5, nicotine treatment not only increased the co-localized spots of p97 with EEA1 (Figure 5a,b) but also decreased the co-localized spots of p97 with calnexin (Figure 5c,d). As EEA1 and calnexin are the markers of early endosomes and ER, respectively [22], these data indicate that the treatment with nicotine promote the endosomal relocation of p97 from ER. Importantly, K48R-Ub abolished nicotine’s effect on the endosomal relocations of p97 (Figure 5). Given that K48R-Ub efficient inhibited nicotine-augmented K48-linked ubiquitination of MR (Supplementary Figure S3), above data indicate that K48-linked ubiquitination of MR is essential for nicotine-increased relocation of p97 from the ER toward the endosomes.

### 3.6. K48-Linked Ubiquitination Contributes to Nicotine-Increased Endosomal Relocation of Sec61 in Dendritic Cells

**Figure 6:**
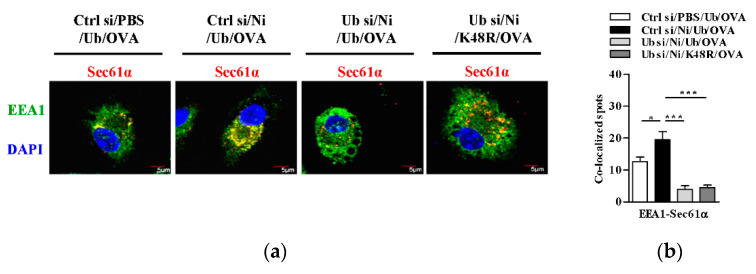

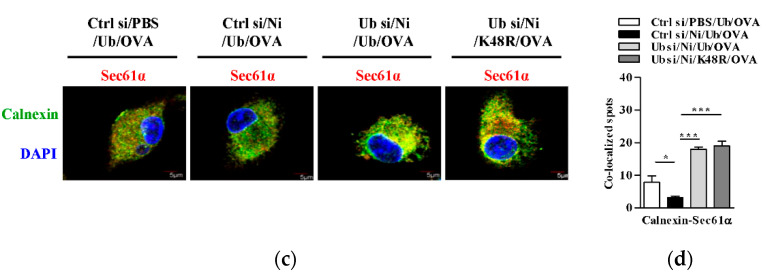
K48-linked ubiquitination contributes to nicotine-increased endosomal relocation of Sec61α in dendritic cells. (**a**,**b**) Murine ubiquitin-deficient and control BM-DC were stimulated with nicotine (10^−7^ mol/L) for 12~16 h. Then, the cells were replenished with K48R-Ub or WT-Ub (30 × 10^−6^ mol/L) and further incubated with ovalbumin (50 μg/mL) for 5~6 h. The endosomal relocation of Sec61α (**a**) from the endoplasmic reticulum (**b**) was determined by confocal microscope with EEA1, calnexin, and Sec61α antibody staining. EEA1 (**a**), calnexin (**b**) were stained green; Sec61α was stained red; nuclei were counterstained with DAPI (blue). The co-localized spots of Sec61α with EEA1 (**c**), Sec61α with calnexin (**d**) were counted and analyzed. Data are presented as the mean ± SEM, * *p* < 0.05, *** *p* < 0.001, one-way ANOVA with Newman-Keulspost test. One representative from three independent experiments is shown. EEA1: early endosome marker; Calnexin: endoplasmic reticulum marker; Ni: nicotine; Ub: ubiquitin; si: siRNA; OVA: ovalbumin.

It was documented that Sec61, another protein in the ER, translocate toward endosomes upon antigen loading and provide energy for transmembrane movement of the antigen [17,18,19]. To investigate the potential role of K48-linked ubiquitination in nicotine-augmented DC cross-presentation, we incubated BM-DC with K48R-Ub and assessed the co-localized spots of Sec61α with EEA1 or with calnexin. As shown in Figure 6, nicotine treatment augmented the co-localized spots of Sec61α with EEA1 (Figure 6a,b) and attenuated the co-localized spots of Sec61α with calnexin (Figure 6c,d). As EEA1 and calnexin stand for early endosomes and ER, respectively [22], these data indicated that the treatment with nicotine facilitate the endosomal relocation of Sec61α from the ER. Importantly, nicotine-affected the co-localized spots of Sec61α with EEA1 (Figure 6a,b) and of Sec61α with calnexin (Figure 6c,d) were completely inhibited by K48R-Ub incubation. Given that K48R efficiently inhibited nicotine-augmented K48-linked ubiquitination of MR (Supplementary Figure S3), these data indicate that K48-linked ubiquitination of MR play important roles in nicotine-increased endosomal relocation of Sec61 in BM-DC.

### 3.7. Nicotine-Increased K48-Linked Ubiquitination of Mannose Receptor in Dendritic Cells

**Figure 7:**
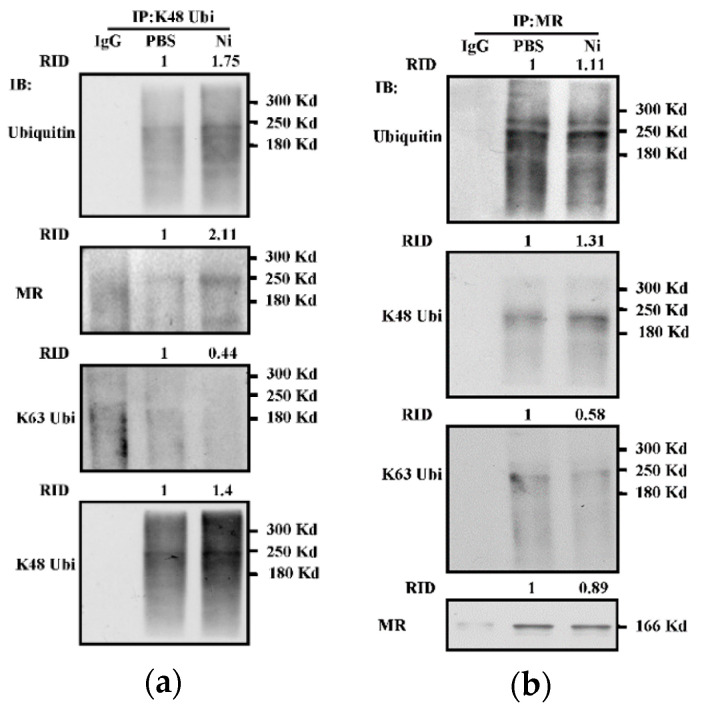
Nicotine increases K48-linked ubiquitination of mannose receptor in dendritic cells. (**a**,**b**) Murine BM-DC was stimulated with nicotine (10^−7^ mol/L) for 12~16 h. Whole cellular protein was extracted and Co-IP assay was performed with mannose receptor (**a**) or K48-linked ubiquitination (**b**) antibody. The levels of mannose receptor, ubiquitination, K48-linked and K63-linked ubiquitination were determined by Western blot analyses. Protein level was quantified by ImageJ software and presented as related integrated density (RID). One representative from three independent experiments is shown. Ni: nicotine; K48 Ubi: K48-linked ubiquitination; K63 Ubi: K63-linked ubiquitination; MR: mannose receptor; IP: immunoprecipitation; IB: immunoblotting.

The mechanism of antigen uptake determined the entrance of antigens into a specific intracellular pathway, which is required for efficient cross-presentation [26]. MR internalized OVA targets early endosomes and inhibits endosomes’ maturation [18,21]. As MR is an important receptor for DC cross-presentation [21] and K48-linked ubiquitination facilitates nicotine-increased endosomal relocation of p97 and Sec61, we wonder whether nicotine-increased cross-presentation is attributed to K48-linked ubiquitination of MR. As shown in Figure 7a, an obvious interaction between MR and K48-linked ubiquitination can be observed in the output of nicotine-treated K48-link ubiquitin antibody anticipated Co-IP (Figure 7a). Meanwhile, an increased interaction between K48-linked ubiquitination and MR can also be monitored in the output of MR antibody-anticipated Co-IP (Figure 7b). Interestingly, K48R-Ub replenishment efficiently abrogated the effect of nicotine on K48-linked ubiquitination (Supplementary Figure S5). Importantly, an increased interaction between K63-linked ubiquitinated protein and MR cannot be achieved (Figure 7b). All these data indicate that the treatment with nicotine increased K48-linked ubiquitination of MR in BM-DC. 

## 4. Discussion

In this study, we investigated the roles of K48-linked ubiquitination in nicotine-increased BM-DC-mediated adaptive immunity by observing cross-presentation, cross-priming, the endosomal recruitments of p97 and Sec61, and the cytolytic activities of DC-activated T cells. We found that K48-linked ubiquitination contributes to nicotine-increased endosomal recruitments of p97 and Sec61, leading to nicotine-augmented cross-presentation, cross-priming, and thereby the capacity of cytotoxicity. Importantly, nicotine-increased BM-DC cross-presentation is attributed to K48-linked ubiquitination of MR (Supplementary Figure S6). Whereas nicotine induces bronchial epithelial cell apoptosis and senescence ROS-mediated autophagy [13], nicotine also increases plasma cell abundance after immunization [3]. Recently, we demonstrated that the combined action of up-regulation of MR enhanced the endosomal recruitment of TAP contributing to nicotine-increased cross-presentation [21]. In the present study, we further demonstrated that K48-linked ubiquitination contributes to the superior adaptive immunity of nicotine-administrated BM-DC. Our study provides a new insight into the mechanism of nicotine-augmented cross-presentation, which might thus open new opportunities for therapeutic intervention of DC-based T-cell vaccine.

Ubiquitination regulates the degradation, membrane trafficking, and transcription of proteins [9]. The exposure of nicotine inhibits proteasomal activities and decreases the degradation of α7 nAChR, indicating that the degradation of α7 nAChR is proteasome-dependent [12]. The activation of α7 nAChR induces the degradation of I-kappa B Kinase α (IκBα) and results in the phosphorylation and nuclear accumulation of nuclear factor kappa-B (NF-κB) p65 [27]. The inhibition of NF-κB pathway was found to contribute to PYR-41 impaired cross-presentation [22]. Moreover, TLR4-MyD88-IRAK4 pathway facilitates nicotine-increased cross-presentation by augmenting the endosomal relocation of TAP [21]. Hence, nicotine-increased DC-mediated adaptive immunity might be attributed to α7 nAChR accumulation and the endosomal relocation of components of cross-presentation machinery [21].

K48-linked and K63-linked ubiquitination regulate proteolytic and signaling pathways, respectively [28]. K48-linked ubiquitination was reported to protect K63-linked ubiquitination from cylindromatosis (CYLD)-mediated deubiquitylation, thereby amplifying NF-κB signals [29]. Our previous findings show that the inhibition of NF-κB activation impairs DC cross-presentation [19,22], indicating that NF-κB plays a vital role in DC cross-presentation. In the present study, K48-linked ubiquitination was verified to contribute to nicotine-increased cross-presentation. As α7 nAChR interacts with protein phosphatase-1γ (PP1γ) and facilitates the activation of TNF receptor-associated factor 6 (TRAF6) [27], the role of K48-linked ubiquitination in α7 nAChR accumulation-induced NF-κB activation still needs further investigation. Whereas, nicotine reduces proteasomal activity and produces the accumulation of ubiquitinated proteins [30], c-Cbl was also documented to increase K48-linked ubiquitination of TRAF6 [31]. Hence, the mechanism nicotine-increased K48-linked ubiquitination of MR might be attributed to either the inhibitory effect of nicotine on proteasome or the catalyzing function of c-Cbl. 

Here, we provided evidences that K48-linked ubiquitination is essential for nicotine-increased endosomal recruitments of p97 and Sec61, indicating that nicotine-increased cross-presentation can be restricted to antigen-containing endosomes. Whereas Akt^+^IKKα/β^+^Rab5^+^ signalosomes contribute to the endosomal recruitment of Sec61 [19], ubiquitinated MR is also important for the relocation of p97 toward endosomes [18]. UBXN2A, a p97 adaptor protein that facilitates the trafficking of α3-containing nAChR, interacts with carboxyl terminus of Hsc70 interacting protein (CHIP) and contributes to α3 ubiquitination [32]. Hence, nicotine-increased endosomal recruitments of p97 and Sec61 might also be attributed to the interactions between UBXN2A and ubiquitinated MR. On the other hand, we also notice that ubiquitin-deficient cells reconstituted with WT-Ub or with K48R-Ub have the similar behaviors in the endosomal relocations of Sec61 and p97 (Figure 5 and Figure 6), which indicate that the replenishment with WT-Ub cannot reconstitute nicotine-increased K48-linked ubiquitination of MR. Whereas the inhibition of dopamine receptor D3 signaling in DC increases antigen cross-presentation [33], dopamine-dependent behavioral effects of nicotine were also documented [34]. As nicotine treatment inhibits proteasomal activities [12] and increased dopamine release [35], the phenomena that WT-Ub fails to restore the ubiquitination-induced effects might be due to nicotine-increased dopamine release and the potential role of dopamine receptor D3 signaling in DC cross-presentation. 

## 5. Conclusions

K48-linked ubiquitination of MR contribute to the endosomal recruitment of p97/Sec61 and the superior cross-presentation efficacy of BM-DC.

## Data Availability

The data presented in this study are available on request from the corresponding author.

## Supplementary Materials

The following are available online at https://www.mdpi.com/2076-393X/9/3/278/s1. Figure S1: The treatment with nicotine increases K48-linked ubiquitination in bone marrow-derived dendritic cells. Figure S2: Ubiquitin siRNA transfection efficiently decreases ubiquitination in dendritic cells. Figure S3: K48R ubiquitin replenishment attenuates nicotine-increased K48-linked ubiquitination of mannose receptor in dendritic cells. Figure S4: K48-linked ubiquitination contributes to nicotine-increased cross-presentation in dendritic cells. Figure S5: K48R ubiquitin replenishment specific inhibits nicotine-increased K48-linked ubiquitination in dendritic cells. Figure S6: Model of K48-linked ubiquitination contributes to nicotine-increased cross-presentation in dendritic cells.
