# Supplementary material for: K48-Linked Ubiquitination Contributes to Nicotine-Augmented Bone Marrow-Derived Dendritic-Cell-Mediated Adaptive Immunity
Source: Vaccines (Basel). 2021 Mar 19;9(3):278. doi: 10.3390/vaccines9030278 (PMC8003133; doi:10.3390/vaccines9030278)
Supplement: Supplementary file 1 [file vaccines-09-00278-s001.pdf]

Supplementary figure legend

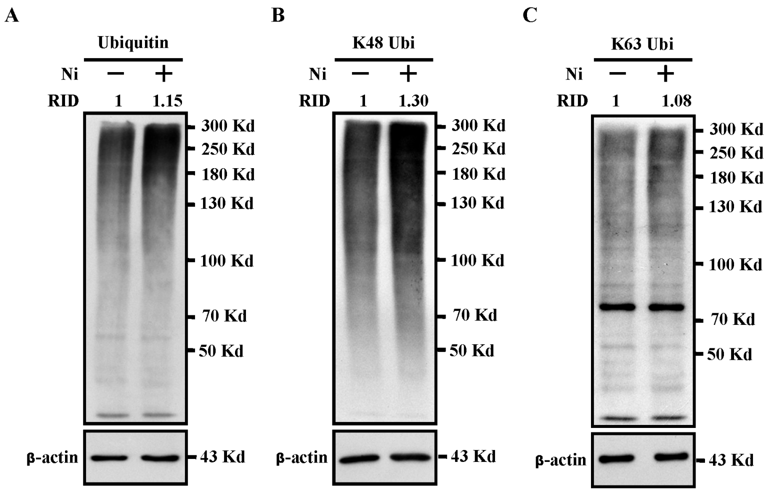

Figure S1: The treatment with nicotine increases K48-linked ubiquitination in bone marrow precursor cell-derived dendritic cells. (a-c) Murine GM-CSF and IL-4 treated BM-DC was incubated with nicotine (10^{-7} mol/L) for 12~16 h with MG132 (50×10^{-6} mol/L). The effects of nicotine on the ubiquitin chains (a), K48-linked ubiquitination (b) or K63-linked ubiquitination (c) were determined by Western blot analyses. β-actin was used as an internal control. Protein level was quantified by ImageJ software and presented as related integrated density (RID). One representative from 3 independent experiments is shown. Ni: nicotine; K48 Ubi: K48-linked ubiquitination; K63 Ubi:K63-linked ubiquitination.

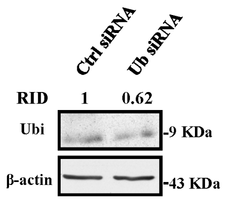

Figure S2: Ubiquitin siRNA transfection decreases ubiquitination in dendritic cells. Murine GM-CSF and IL-4 treated BM-DC were conferred with ubiquitin or control siRNA transfection at the final concentration of 50 pmol/L. Then whole cellular protein was extracted and the level of ubiquitination in the cells was determined by Western blot analyses. Protein level was quantified by ImageJ software and presented as related integrated density

(RID). Ubi: ubiquitin.

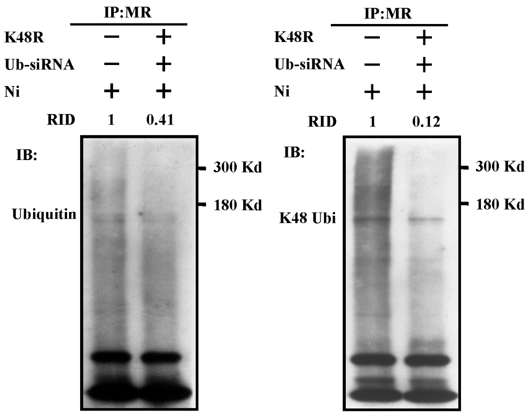

Figure S3: K48R ubiquitin replenishment attenuates nicotine-increased K48-linked ubiquitination of mannose receptor in dendritic cells. Murine ubiquitin deficient and control BM-DC were incubated with nicotine (10^{-7} mol/L) and K48R or control ubiquitin peptide (30×10^{-6} mol/L) for 12~16 h with MG132 (50×10^{-6} mol/L). The levels of ubiquitination and K48-linked ubiquitination of mannose receptor (MR) was determined by western blot in MR antibody-anticipated Co-IP. Protein level was quantified by ImageJ software and presented as related integrated density (RID). Ni: nicotine; Ub: ubiquitin; MR: mannose receptor; K48 Ubi: K48-linked ubiquitination; IP: immunoprecipitation; IB: immunoblotting.

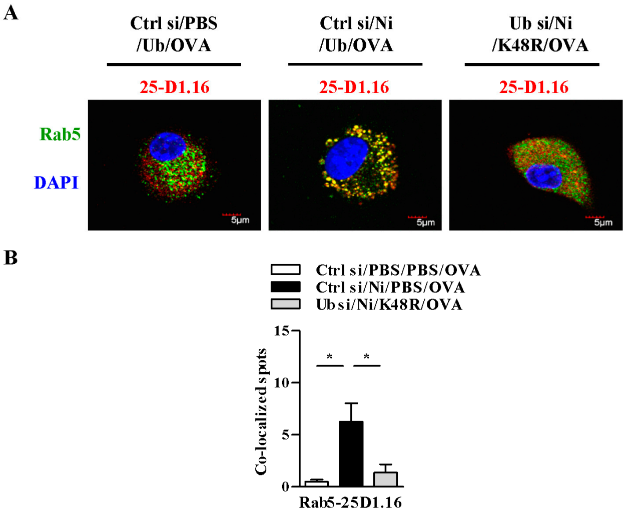

Figure S4: K48-linked ubiquitination contributes to nicotine-increased cross-presentation in dendritic cells.

(a-b) Murine ubiquitin deficient and control BM-DC were incubated with nicotine (10^{-7} mol/L) and K48R or control ubiquitin peptide (30×10^{-6} mol/L) for 12~16 h. Then, the cells were further incubated with ovalbumin (50 μg/mL) for 5~6 h. Cross-presented OVA was assessed by confocal microscope (a-b). Cross-presented OVA was stained with 25-D1.16 (red) and Rab5 was stained with green (a) respectively; nuclei were counterstained with DAPI (blue). The co-localized spots of 25-D1.16 with Rab5 were counted and analyzed (b). Data are presented as the mean±SEM, *$p<0.05$, one-way ANOVA with Newman-Keulspost test. One representative from 3 independent experiments is shown. Ni: nicotine; Ub: ubiquitin; si: siRNA; Rab5: early endosome marker; 25D1.16: antibody specific to SIINFEKL-H2Kb complex; OVA: ovalbumin.

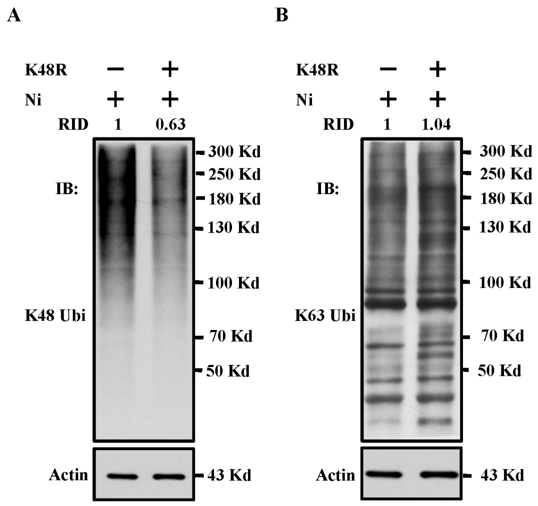

Figure S5: K48R ubiquitin replenishment specific inhibit nicotine-increased K48-linked ubiquitination in dendritic cells. Murine ubiquitin deficient and control BM-DC were incubated with nicotine (10^{-7} mol/L) and K48R or control ubiquitin peptide (30×10^{-6} mol/L) for 12~16 h with MG132 (50×10^{-6} mol/L). The levels of K48-linked ubiquitination (a) and K63-linked ubiquitination (b) in the cells were determined by Western blot analyses. Protein level was quantified by ImageJ software and presented as related integrated density (RID). Ni: nicotine; Ub: ubiquitin; K48 Ubi: K48-linked ubiquitination; K63 Ubi: K63-linked ubiquitination; IB: immunoblotting.

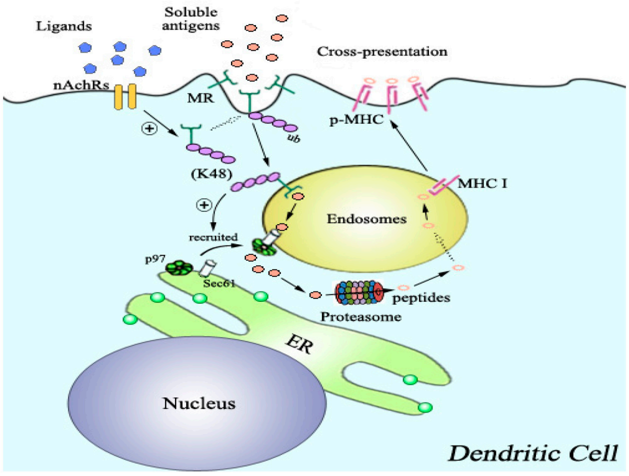

Figure S6: Model of K48-linked ubiquitination contributes to nicotine-increased cross-presentation in dendritic cells. K48-linked ubiquitination of mannose receptor, together with the endosomal recruitments of p97 and Sec61, facilitate nicotine-increased BM-DC cross-presentation, cross-priming, and subsequent the cytolytic activities of DC-activated T cells.
